# Development of a 'toolkit' to identify medical students at risk of failure to thrive on the course: an exploratory retrospective case study

**DOI:** 10.1186/1472-6920-11-95

**Published:** 2011-11-18

**Authors:** Janet Yates

**Affiliations:** 1 Medical Education Unit, B94 Medical School, Queen's Medical Centre, Nottingham NG7 2UH, UK

## Abstract

**Background:**

An earlier study at Nottingham suggested that 10-15% of the medical student intake was likely to fail completely or have substantial problems on the course. This is a problem for the students, the Faculty, and society as a whole. If struggling students could be identified early in the course and additional pastoral resources offered, some of this wastage might be avoided. An exploratory case study was conducted to determine whether there were common indicators in the early years, over and above academic failure, that might aid the identification of students potentially at risk.

**Methods:**

The study group was drawn from five successive cohorts. Students who had experienced difficulties were identified in any of four ways: from Minutes of the Academic Progress Committee; by scanning examination lists at key stages (end of the first two years, and finals at the end of the clinical course); from lists of students flagged to the Postgraduate Deanery as in need of extra monitoring or support; and from progress files of those who had left the course prematurely. Relevant data were extracted from each student's course progress file into a customised database.

**Results:**

1188 students were admitted over the five years. 162 (14%) were identified for the study, 75 of whom had failed to complete the course by October 2010. In the 87 who did graduate, a combination of markers in Years 1 and 2 identified over half of those who would subsequently have the most severe problems throughout the course. This 'toolkit' comprised failure of 3 or more examinations per year, an overall average of <50%, health or social difficulties, failure to complete Hepatitis B vaccination on time, and remarks noted about poor attitude or behaviour.

**Conclusions:**

A simple toolkit of academic and non-academic markers could be used routinely to help identify potential strugglers at an early stage, enabling additional support and guidance to be given to these students.

## Background

Every year, a small number of undergraduate students at the University of Nottingham Medical School fail to make satisfactory progress on the course. Some have problems at all stages, others have sporadic difficulties, and a proportion fail to graduate at all, either leaving voluntarily or having their course terminated. In an earlier study we found that 10-15% of the annual intake were affected to some degree [1]. There is often a combination of causative factors, including difficulty in coping with the academic workload, health problems, and social issues such as not settling at university or having adverse personal or family circumstances. Some students may have applied for medicine as a result of family pressures rather than personal choice and may therefore lack motivation, or have unrealistic expectations.

Failure to thrive on the course, or 'struggling' in the case of the worst-affected, is a cause for concern. Firstly it is a concern for the student, who may suffer considerable personal distress as a result of exam failures or poor health, with possible financial hardship if their course has to be extended, and perhaps stigma or shame after total failure. Secondly there is an increased load on the Faculty and the university, with a disproportionate amount of time spent on meeting and advising the struggling students, setting additional exams, and in some cases dealing with Fitness to Practise hearings or Appeals against termination. Thirdly, there is a societal cost attached to the student who drops out after receiving public funding. There is evidence that some poor students may become poor doctors, subsequently failing in professional life [2-4]. Strenuous attempts are therefore made to support and advise students in difficulty.

The admissions process at Nottingham has been developed and refined over the years in order to select those whom we feel have the best chances of becoming good doctors and to de-select those who are felt to be unsuitable candidates. To this end, there is a 4-part process: screening of UCAS forms for a minimum academic standard; an online, computer-marked questionnaire to explore the candidate's extra-curricular activities and aptitudes; screening of the personal statement by experienced Faculty staff, to check for factors such as work experience; and finally a semi-structured interview which enquires about motivation, empathy, and communication skills. Despite these measures, problems still occur, ranging from the students who decide within weeks that they do not want to study medicine after all, to those who battle on through an extended course of six, seven or occasionally eight years.

All medical students at Nottingham have the benefit of a comprehensive pastoral care system. They each have a personal tutor who heads a 'Medical Family' of two to four students from each year group. The student will have a 'parent' within the Medical Family, a student in the year above them, to provide close peer support. The tutor and student meet formally at pre-arranged intervals to monitor overall progress and discuss any problems in confidence, and the student may also contact the tutor at other times for informal discussions. Further advice and support can be sought from the Senior Tutors, or Associate Dean for Medical Education. Those who fail exams will be seen at the Academic Progress Committee for support, or can be referred, or self-refer, to the Clinical Sub-Deans for personal mentoring. Students can also be directed towards sources such as the Occupational Health Service or University Counselling Service, and of course their GP or other external agencies. They are regularly reminded to seek help earlier rather than later, to submit extenuating circumstances forms in the event of acute events which might affect exam performance, and that they must take responsibility for their own conduct and performance [5,6]. However, failure to engage with these supportive mechanisms still occurs and may well contribute to failure to thrive. Reluctance to seek help from medical professionals has been attributed to concerns about confidentiality and the potential longer-term effects on career and reputation [7-10]. Students who lack insight into their own shortcomings may decline to accept feedback and help [11-13] and have the potential to become irremediable doctors [14].

Although academic achievement is known to predict later performance [15-17] and is the easiest to monitor, many other factors are important. Medical students are known to suffer high rates of stress and depression, especially at the start of the course [18,19], and our own research has suggested a high incidence of depressive-type illness in struggling students [20]. Another significant aspect of underperformance is unprofessional behaviour, which is increasingly important for both medical students and practising doctors [6,21]. Although difficult to measure, there is evidence that lapses in behaviour as a student may be associated with poorer performance [22,23] and with deficiencies in later professional life [4]. A low threshold for detecting unprofessional behaviour, and clear strategies for dealing with it, are recommended [24,25]. At Nottingham we have recently introduced a 'Concerns Form' which may be used by students or staff to report unsatisfactory behaviour (see Additional file 1).

It is obviously better for all concerned if difficulties can be identified as early as possible. This would provide the best chance for remedial help, whether that be advice on study skills, time out for recovery and recuperation after illness, or even gently steering a student towards a more appropriate non-clinical career. (The Nottingham undergraduate course includes the award of an integrated BMedSci degree at the end of the third year. This enables students to leave at this point and move into research or other areas of study if they find, after the first six months of the full-time clinical course, that they are not suited to medicine. In some circumstances they can transfer to a BSc degree in Medical Sciences during the third year, avoiding the start of the clinical course entirely).

With this in mind, we decided to conduct a detailed review of the course progress files of students who had started the course between 2000 and 2004 inclusive and were known to have experienced any difficulties. We recorded any factors that might have a bearing on student's progress - academic, health, or social issues, and incidents of adverse attitude or behaviour - to see if there was any reliable combination during the first two years of study which might constitute a toolkit to identify those most at risk.

Ethical approval was granted by the University of Nottingham Research Ethics Committee, ref B/11/2009.

## Methods

The target students were from the cohorts who had entered the course in 2000-2004 inclusive and had demonstrated unsatisfactory progress at any stage or who had left prematurely. Several methods were used to identify these students, none of whom had been included in our previous study [1]:

› The Minutes of the Academic Progress Committee

› Inspection of Part I examination marks (average over Years 1 and 2) for students scoring below 50%, and of Year 5 examination marks for students failing their Finals at first attempt.

› Lists compiled by the Clinical Sub-Deans of students who might require support or additional monitoring at the Foundation Deanery during their first post-graduate year

› Archived files of students who had failed to graduate

The course progress file of every student identified was then searched manually and all potentially relevant data extracted into a customised database. The data included:

› Available pre-admission factors. These were age at course entry, sex, fee status, declared disability, academic qualifications (number of A levels at Grade A, B or other), and type of offer made after interview (unconditional, conditional, reserve, or late).

› Key markers of course progress or difficulty in each year. These included exams or modules failed, extenuating circumstances brought forward, problems with health, disruptive personal/family/social difficulties, attendance at the Academic Progress Committee (APC), and interviews with senior pastoral care staff or Clinical Sub-Deans.

These categories of information were recorded as yes/no options with supplementary text boxes. We did not routinely include actual examination marks but recorded the number of exams failed in Years 1 and 2. Because some students had frequent low marks without actual fails, we did note the mark for Part I, which is a weighted average of all Year 1 and Year 2 summative assessments.

Additional File 2 provides a general description of the course.

› Summary options at the end of each year/course phase up to the end of Year 4 (normal progress, progress with resits, repeat year required, student left the course voluntarily, or course terminated)

› Final summary outcome for the final year (graduated BMBS, graduated after repeat exams, graduates after repeat final year, or failed to graduate)

› Additional fields for any repeated years

› The occurrence of any behaviour or attitudes that might constitute a 'concern' as currently defined by the Faculty, eg inappropriate behaviour to others, lateness or non-attendance at teaching, lack of commitment (see Additional File 1). In view of the findings of Wright & Tanner (2002) and Papadakis et al (2005) we included failure to complete mandatory Hepatitis B vaccinations by the end of Year 2, without undue reminders, as a discrete category of unprofessional behaviour.

› Overall outcome summary. This was a semi-objective classification made after data extraction, and contrasts with our previous paper which used an all-inclusive definition of struggling [[1]. We categorised students as:

○ 'struggler' with multiple problems throughout the course

○ 'preclinical' - problems largely confined to the early years

○ 'clinical' - problems largely confined to the later years

○ 'health-related' - problems largely related to ill health

○ 'borderline performance' - weak student, generally low marks throughout

○ 'no substantial problems'. Some students who were identified, for example, via APC attendance, had actually suffered only a minor or one-off drop in performance, and were subsequently eliminated from the database

○ left the course voluntarily

○ course terminated

Further variables were generated as required during the analysis, to create 'flags', and these are described in the Results section.

### Data analysis

After checking and cleaning of the Access database, the file was transferred into SPSS v17 for descriptive analysis of categorical and yes/no fields. Text fields were handled by drawing up Reports in MS Access which were then scanned visually for indicators of difficulty (eg academic failures, disruptive physical or mental health problems), to generate semi-qualitative data.

In a secondary analysis, the data for students who failed to complete the course were examined, to determine why they left the course or failed to graduate, and this will be reported separately.

## Results

### 1 Students on the database

In total there were 1188 students admitted to the course over the five years. Table 1 illustrates the categorisation of 194 (16%) students identified by the means described above. After elimination of the 32 with no substantial problems, and two still on an extended course, the database contained 87 course-completers and 73 non-completers, representing 7% and 6% of the intake, respectively.

**Table 1:** Broad categories of students identified within the database

Completed the course	n = 87
Struggler - problems in preclinical & clinical parts of course	25
Preclinical problems predominated	18
Clinical problems predominated	8
Problems largely health-related	17
Borderline performance	19
(No substantial problems, discarded from database	32)

**Did not complete the course**	**n = 75**

Still on course, discarded from database	2
Left course voluntarily	59
Course terminated	14

### 2 Socio-demographic characteristics and qualifications

Table 2 shows the socio-demographics of the study group. There were no major differences between the course-completers and non-completers. The proportion of males was slightly higher than in the entire intake (43% compared with 37%), as was the proportion of overseas students (15% compared to 10%), but with small numbers these differences were not statistically significant with Chi-square tests.

**Table 2:** Socio-demographic characteristics of student group identified for the study

		Course completers		Non-completers		Combined	
		n	% *	n	% *	n	% *
Domicile	Home	74	85	64	85	138	85
	EU	0	0	0	0	0	0
	Overseas	13	15	11	15	24	15
							
Sex	Male	39	45	30	40	69	43
	Female	48	55	45	60	93	57
							
Age at course entry	Under 21	82	94	71	95	153	94
	21 or over	5	6	4	5	9	6
							
Any disability declared	Yes	4	5	1	1	5	3
	No	83	95	74	99	157	97
							
School leaver	Yes	83	95	72	96	155	96
	No	4	5	3	4	7	4
							
Interview offer type	Unconditional	5	6	6	8	11	7
	Conditional (on A levels)	71	82	59	79	130	80
	Reserve	4	5	2	3	6	4
	Late offer (May-August)	7	8	7	9	14	9
	Not known	0	0	1	1	1	0.5

* percentage figures may not total 100 due to rounding up.

As expected, the majority of students (145/162, 90%) were aged 18-20 at course entry, and the median age of the group was 18. Nine students (5%) were only 17, of whom five completed the course, and nine (5%) were over 21, again with five being completers.

A-level data were available for 149 (92%) of the students, and of these, 127 (85%) had at least 2 'A' grades, as would normally be required for acceptance on the course. There was no suggestion that the non-completers were less well qualified, in terms of A-levels.

### 3 Overall outcomes

**Figure 1:**
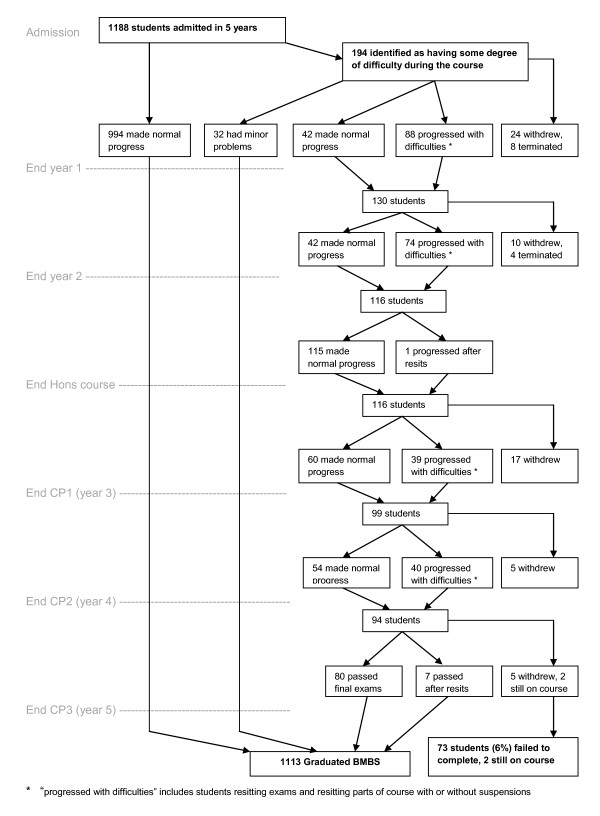
**Summary of course progress and attrition on the 5-year undergraduate medical course**.

Figure 1 shows the overall outcomes, in terms of normal or abnormal progress and attrition, at each stage of the course. The first two years are largely preclinical in content. The third year is split between the Honours course (individual projects plus taught courses) and the early clinical course (Clinical Practice 1, CP1). The final two years encompass the clinical course, CP2 and CP3. As can be seen from the Figure, the largest proportion of course exits occurred during the first two years, but an appreciable number of students fail during the later years. Within those who graduated, there were 29 who repeated at least one year of the course due to academic failure or ill health.

### 4 Potential early markers of students who fail to thrive

Analysis focused on the first two years for the 87 students who did complete the course despite varying degrees of difficulty. After a thorough review of the database we selected categories of underperformance or concern. The markers that we chose, duplicated for each year, were:

• Failure in three or more modular examinations - students sit 10 assessments in each of the first two years, mostly computer-marked multiple-choice or short answer formats, but including some essay, course-work, or other formats. It is not unusual for students to fail one or perhaps two components, but three or more would certainly be a cause for concern.

• An overall mark <50% for all summative assessments in Year 1 and 2 (Part I). 50% is the minimum requirement for onward progression to the BMedSci.

• Poor attendance - any mention in the progress file of the student missing mandatory teaching or a scheduled meeting without prior permission or explanation.

• Unprofessional behaviour - failing to communicate with staff or tutors, failing to respond to Faculty emails, challenging Faculty decisions with an adversarial attitude, displaying arrogant or flippant attitudes, failing to disclose other relevant information such as illness. External events involving the police also came into this category.

• Health problems - documentation of ongoing or recurrent illness which was clearly affecting the student's ability to function on the course. Anxiety and depression were the most common. Acute illnesses such as viral infections were not included.

• Social problems - documentation of factors such as unsatisfactory housing, family pressures, or overseas family commitments, which clearly impacted on students' progress.

• Failure to complete Hepatitis B vaccination schedules on time.

We totalled this selection of 'flags' for the entire group and also by the overall outcome (struggler, borderline etc), as shown in Table 3. It is clear that those whom we had classified as strugglers on the basis of their performance throughout the course had a high incidence of early academic failure, as did the preclinical group. The latter had the highest proportion of social problems, whereas the strugglers seemed more likely to have had a poor attendance record. However, with these small numbers of students, individual flags might not be as useful as a combination of them all.

**Table 3:** Flags for academic and non-academic difficulty in those who completed the course

	Number (%) of students in each group acquiring each type of flag					
	Struggler (preclinical & clinical) N = 25	Preclinical problems only N = 18	Borderline performance N = 19	Clinical problems only N = 8	Problems largely health- related N = 17	All students N = 87
Year 1						
Failed >= 3 assessments	13 (52)	10 (55)	7 (37)	0	5 (29)	35
Poor attendance noted	10 (40)	2 (11)	3 (16)	1 (13)	4 (24)	20
Poor behaviour noted	8 (32)	4 (22)	1 (5)	0	2 (12)	15
Health problems noted	3 (12)	1 (6)	1 (5)	0	3 (18)	8
Social problems noted	3 (12)	5 (28)	1 (5)	1 (13)	1 (6)	11

Year 2						
Failed >= 3 assessments	21 (84)	15 (83)	5 (26)	1 (13)	4 (24)	46
Poor attendance noted	13 (52)	4 (22)	4 (21)	0	3 (18)	24
Poor behaviour noted	6 (24)	0	2 (11)	0	1 (6)	9
Health problems noted	2 (8)	2 (11)	0	0	5 (29)	9
Social problems noted	6 (24)	6 (33)	0	0	0	12
Hepatitis B vaccinations incomplete	4 (16)	0	2 (11)	0	1 (6)	7
Part I mark <50%	9 (36)	7 (39)	6 (31)	0	3 (18)	25

We therefore totalled the flags for each student, for Year 1, and for Year 1 plus Year 2 with or without the flag for <50% in Part I, to see if this might be predictive. The results are shown in Table 4. In Year 1 alone, a total of two or more flags picked out 10/25 (40%) of the strugglers, compared to 5/18 (28%) of the preclinical group, 2/19 (11%) of the borderline group, 5/17 (29%) of those with health-related difficulties, and none of the clinical problems group. In Year 2 alone, two or more flags detected 15/25 (60%) of strugglers, 10/18 (56%) of the preclinical group, 3/19 (16%) of the borderlines, 4/17 (24%) of the health-related group, and again none of the clinical group.

**Table 4:** Total flags in Year 1 and Year 2 by student outcome category

		Struggler (preclinical & clinical) N = 25	Preclinical problems only N = 18	Borderline performance N = 19	Clinical problems only N = 8	Problems largely health- related N = 17	All students N = 87
Total predictive flags in Year 1 only: Failed >= 3, attitude, attendance, health & social	0 flags	7	5	11	6	8	37
	1 flag	8	8	6	2	4	28
	2 flags	3	1	1	0	4	9
	3 flags	5	4	0	0	1	10
	4 flags	2	0	0	0	0	2
	5 flags	0	0	1	0	0	1
							
Number (%) in each group who had two or more flags		10 (40)	5 (28)	2 (11)	0	5 (29)	

Total predictive flags in Year 2 only: Failed >= 3, attitude, attendance, health, social & missed vaccinations	0 flags	1	2	11	7	8	29
	1 flag	9	6	5	1	5	26
	2 flags	5	9	1	0	3	18
	3 flags	7	1	2	0	1	11
	4 flags	3	0	0	0	0	3
							
Number (%) in each group who had two or more flags		15 (60)	10 (56)	3 (16))	0	4 (24)	

Total predictive flags in Year 1 + Year 2: Failed >= 3, attitude, attendance, health, social, missed vaccinations, & Part 1 <50%	0 flags	0	0	7	5	7	19
	1 flag	3	1	2	3	2	11
	2 flags	2	4	5	0	1	11
	3 flags	6	9	3	0	3	21
	4 flags	7	1	0	0	3	12
	5 flags	1	1	2	0	0	4
	6 flags	2	2	0	0	0	4
	7 flags	4	0	0	0	1	5
							
Number (%) in each group who had four or more flags		14(56)	4 (22)	2 (11)	0	4 (24)	

When the Year 1 and 2 flags were added, including the low Part I marker, the distinction became greater. A total of four or more flags then selected 14/25 (56%) of the strugglers, but only 4/18 (22%) of the pre-clinical group, 2/19 (11%) of the borderlines, 4/17 (23%) of the Health-related group, and again none of the clinical problems group.

We would not expect that students making largely uneventful progress would acquire four or more flags. We checked the files of 12, chosen at random from the 32 categorised as 'no substantial problems'. At the end of Year 2, five had no flags, and four had one (one failed 4 exams in Year 1, no further problems and 2.1 degree; one borderline 49% at end of year 2; one failed 3 exams in Year 1; and one had a flag for attitude in Year 1). One had a flag for attitude in Year 1 and for attendance in Year 2. Two students had three flags and in retrospect should perhaps have been classified as 'borderlines', but none had four or more.

### 5 The non-completers

The 75 students who did not graduate will be the subject of a separate paper. They could not be assessed by the same combinations of flags, for the following reasons:

• Over half (46/75, 61%) left within the first two years, many within the early weeks or months, and sometimes withdrawing before completing their examinations. These students had no academic flags and their files held little or no information on their personal circumstances.

• A further 16 (21%) left at the end of year 3, when they had their BMedSci; some were academically very able students who chose to move elsewhere for their clinical studies, others had simply decided that medicine was not for them. These choices are unlikely to be predictable in any way.

• A third group (11/75, 15%) who left later in the course, were predominantly beset by mental health problems and most had not had difficulties in the early years. Only one could have been described as a struggler and in fact had six flags at the end of Year 2.

• As noted, two students had not completed their course so were excluded from analysis.

## Discussion

Our study suggests that routine monitoring of a number of simple criteria could provide a good means of identifying many of the students potentially at risk of struggling. Although early academic failure may be the first sign of trouble, the consideration of non-academic criteria adds a wider dimension and demonstrates the value of recording adverse health, social and behavioural events. This standardised toolkit will enable early remedial action to be taken by means of targeted academic and pastoral support, which could include guidance on alternative careers.

The way in which we defined the different categories of students is obviously a combination of objective and subjective classification and there are no firm dividing lines between, say, a struggler and a borderline student. In addition, the problems experienced are many and varied. We would therefore not expect to find a totally fail-safe means of detecting all potential strugglers. Nevertheless, a workable system for the early detection and remediation of high-risk students may be valuable. There is evidence that the majority of students do welcome feedback and support, but that it must be delivered carefully and is labour-intensive for the Faculty [26]. Academic remediation in group situations may help to overcome the stigma of failure [13], but tailored individual support may be required for specific problems with language, communication and inter-professional skills [27]. Faculty staff at Nottingham already strive to offer this type of support, but reaching those students who resist help remains difficult [11].

It was interesting that we identified a number of students, the 'preclinical' group, whose academic difficulties were at least as severe as the strugglers' in the first two years but who subsequently recovered and performed well in the later course. They appeared more likely to be female and less likely to exhibit poor attitude or behaviour. We have no ready explanation for this finding, which perhaps reflects personality differences in terms of insight and readiness to change, or even the motivation engendered by patient contact and ward-based work.

The 'health-related' group displayed little evidence of underperformance in the early course but developed significant problems, largely mental ill-health, during the clinical course. Several disclosed a long-standing history of anxiety, depression or eating disorders at this stage. The Faculty encourages discussion of such problems both before admission and on the course, including confidential consultations with the Occupational Health service. It is hard to see what else could be done to avoid this late-stage distress. Similarly, the 'clinical problems' group were undetectable in the early course.

## Limitations of the study

This is a relatively small study in a single medical school and the results may not be fully generalisable to other schools with different curricula, teaching styles and student profiles. However, medical education literature from the UK and elsewhere suggests that 'struggling' is not unique to Nottingham [11,13,26,28]. We would suggest that a combination of selected academic and non-academic markers might be suitable for other schools who wish to identify potentially high-risk students, and could be a better predictor than academic criteria alone. The inclusion of categories of unprofessional behaviour is an important part of student monitoring. The medical school's new Concerns form will aid this type of data acquisition at Nottingham.

Data collection was limited to what was written in the progress files, so was not necessarily complete, and required subjective evaluation. However, the author has no personal knowledge of, or contact with, any of the students so was able to extract information without prejudice. Students are encouraged to share personal problems with their tutors in confidence, and may be advised to disclose serious adverse events via extenuating circumstance forms. If they choose not to, and their academic progress is largely unaffected, no record will be made. It is therefore likely that many, but not all, of such events are recorded in the progress files. We intend to develop a searchable system for recording such information as is disclosed, but the student's right to confidential discussion must remain. The student who can cope with unexpected events is not a cause for concern.

We specifically designed an observational case study, so did not investigate the files of the non-problematic students in the cohorts and could not calculate statistical comparisons. A full case-control study would be required for this. However, we are confident that students who were making uneventful progress would not acquire more than one or two flags at most, and probably none. By definition, such students would not have academic flags, nor personal or health problems that they could not cope with, otherwise they would have been identified for the study. The brief review of a sample of 12 students who were initially identified but later discarded as having 'no substantial problems' lends weight to this conclusion, although is not definitive proof. A prospective study is planned in order to validate the proposed toolkit.

We could not explore the role of ethnicity in this study since data were not routinely available for the earlier cohorts. However, a number of those with the greatest problems were overseas students, mostly from Asian or African countries. Some had complex issues including language and communication difficulties compared to their UK peers, loneliness and isolation in the UK, family pressures at home, and cultural reticence in admitting to difficulties and being able to engage with help. A recent review and meta-analysis has shown that ethnic minority status has a widespread negative influence on the performance of both medical students and doctors in the UK [29], and requires equally widespread investigation.

We chose to record exam failures rather than actual marks, other than the Part 1 average. We are now looking at a slightly different scheme - a Red Flag for marks below 50% (which would include fails below the standard-set pass mark of 40%) and a Yellow Flag for low marks (50-55%). This might be better for the detection of those who 'bump along the bottom' without absolute failure.

## Conclusions

A simple toolkit of academic and non-academic markers could be used routinely to identify potential strugglers at an early stage, enabling additional support and guidance to be given to these students.

## Competing interests

The author declare that she has no competing interests.

## Authors' contributions

The author designed the study, collected and analysed the data, and wrote the paper.

## Author information

JY is a Research Fellow in Medical Education, working and publishing with David James (formerly Foundation Director of Medical Education at Nottingham) since 2003, and with a particular interest in students who fail to thrive.

## Pre-publication history

The pre-publication history for this paper can be accessed here:

http://www.biomedcentral.com/1472-6920/11/95/prepub

## Supplementary Material

Additional File 1 **This form has been in use over the last couple of years and enables staff or students to raise valid concerns about unsatisfactory or unprofessional behaviour or attitude witnessed or experienced**. Click here for file

Additional File 2 **This document summarises the overall course structure of the 5-year Undergraduate course at the University of Nottingham**. Click here for file
