# Supplementary material for: Development of a 'toolkit' to identify medical students at risk of failure to thrive on the course: an exploratory retrospective case study
Source: BMC Med Educ. 2011 Nov 18;11:95. doi: 10.1186/1472-6920-11-95 (PMC3229499; doi:10.1186/1472-6920-11-95)
Supplement: Additional File 2 — This document summarises the overall course structure of the 5-year Undergraduate course at the University of Nottingham. [file 1472-6920-11-95-S2.DOC]

**Additional File 2: Course structure**

Although some details of the curriculum and course examinations have varied over the period of this study, the overall structure has remained the same. The Assessment and Programme Specification document for 2005-06, for example, states that:

*“The first thirty-six months of the course consists of two years (four semesters) covering early clinical experience and the basic medical sciences followed by an extended fifth semester occupied by Honours level taught courses and a research project. The sixth semester is a bridging period into the Clinical Course (see below) comprising a Clinical Practice Course and a Community Follow-up Project which contributes to the BMedSci degree.*

*The overall strategy for education in this period is a transition from clinically contextualised lectures and practical-based teaching in Years 1 and 2, supplemented with early clinical experience and problem-based learning (PBL), to self-directed research-based learning. In Years 1 and 2 a systems-based approach integrated with early clinical experience and an explicit basic skills curriculum is used. There is also substantial public health and epidemiology input.*

*Months 31-60 comprise the Clinical course. The aim of this part of the course is to further develop core knowledge in the common conditions in the fields of medicine and surgery. During the final two years students will continue to develop their clinical skills and knowledge to apply their learning to the management of disease. Ward-based and clinical teaching will be supported by lectures, seminars and tutorials, and web-based e-learning resources. A logbook outlining the aims and objectives of the clinical attachments will support teaching in each specialty. Integrated into each attachment is the teaching of personal and professional development, clinical pharmacology, pathology and public health medicine. Students will normally also undertake a nine-week elective period providing an opportunity to gain an insight into medical practice in a different environment or culture. The programme will conclude with the Preparation for Pre-Registration House Officer course.”*

Throughout the course there are written examinations in a variety of formats, including multiple choice (MCQ) variants, short answers, short essays or longer coursework essays. The research element in Semester 5 includes a Dissertation and Viva. Practical examinations in the early years include basic clinical and consultation skills, whilst in the later course there are objective structured clinical examinations (OSCEs) for each attachment. At the end of the course there are final written and practical examinations.

A weighted average is calculated from all written and practical assessments in the first two years and constitutes ‘Part1’, as utilised in this study.
